# Supplementary material for: Structure-guided glyco-engineering of ACE2 for improved potency as soluble SARS-CoV-2 decoy receptor
Source: eLife. 2021 Dec 20;10:e73641. doi: 10.7554/eLife.73641 (PMC8730730; doi:10.7554/eLife.73641)
Supplement: Transparent reporting form [file elife-73641-transrepform1.pdf]

eLIFE
1st Floor
24 Hills Road
Cambridge CB2 1JP, UK
P 01223 855340
W elifesciences.org
T @elife

eLife's transparent reporting form

We encourage authors to provide detailed information *within their submission* to facilitate the interpretation and replication of experiments. Authors can upload supporting documentation to indicate the use of appropriate reporting guidelines for health-related research (see EQUATOR Network), life science research (see the BioSharing Information Resource), or the ARRIVE guidelines for reporting work involving animal research. Where applicable, authors should refer to any relevant reporting standards documents in this form.

If you have any questions, please consult our Journal Policies and/or contact us: editorial@elifesciences.org.

Sample-size estimation

- You should state whether an appropriate sample size was computed when the study was being designed
- You should state the statistical method of sample size computation and any required assumptions
- If no explicit power analysis was used, you should describe how you decided what sample (replicate) size (number) to use

Please outline where this information can be found within the submission (e.g., sections or figure legends), or explain why this information doesn't apply to your submission:

N/A; no sample-size estimation or power analysis was used.

Replicates

- You should report how often each experiment was performed
- You should include a definition of biological versus technical replication
- The data obtained should be provided and sufficient information should be provided to indicate the number of independent biological and/or technical replicates
- If you encountered any outliers, you should describe how these were handled
- Criteria for exclusion/inclusion of data should be clearly stated
- High-throughput sequence data should be uploaded before submission, with a private link for reviewers provided (these are available from both GEO and ArrayExpress)

Please outline where this information can be found within the submission (e.g., sections or figure legends), or explain why this information doesn't apply to your submission:

eLife Sciences Publications, Ltd is a limited liability non-profit non-stock corporation incorporated in the State of Delaware, USA, with company number 5030732, and is registered in the UK with company number FC030576 and branch number BR015634 at the address 1st Floor, 24 Hills Road, Cambridge CB2 1JP | August 2014

All experiments were performed at least twice. A respective statement is included in the legends of Figs. 5, 6, 7, 8, 9 and 10.
Biological replicates (virus neutralization assays): separate wells of Vero E6 cells infected with a given infectious dose of SARS-CoV-2 in the absence or presence of a given concentration of ACE2-Fc, with the amount of viral RNA produced during infection being quantified by RT-qPCR.
Technical replicates (e.g. ACE2 activity assays): separate wells containing a given amount of ACE2 and a fluorescent ACE2 substrate, with continuous monitoring of the increase in fluorescence due to substrate cleavage.
Viral neutralization assays (Figs. 7 and 8): all experiments were performed in three biological replicates and repeated at least once (2-4 independent experiments per ACE2 variant). All DSC and BLI measurements (Figs. 5 and 6) as well as all ACE2 activity assays (Figs. 9 and 10) were performed in at least three technical replicates.
Additional individual experiment replicate information is provided in the Results section, the Materials and Methods section, and the legends to Figs. 5, 6, 7, 8, 9 and 10.
No data were excluded.
No high-throughput sequence data were generated in this study.

Statistical reporting

- Statistical analysis methods should be described and justified
- Raw data should be presented in figures whenever informative to do so (typically when N per group is less than 10)
- For each experiment, you should identify the statistical tests used, exact values of N, definitions of center, methods of multiple test correction, and dispersion and precision measures (e.g., mean, median, SD, SEM, confidence intervals; and, for the major substantive results, a measure of effect size (e.g., Pearson's r, Cohen's d)
- Report exact p-values wherever possible alongside the summary statistics and 95% confidence intervals. These should be reported for all key questions and not only when the p-value is less than 0.05.

Statistical analysis information for each experiment is provided in the Results section, the Materials and Methods section, and the legends to Figs. 5, 6, 7, 8, 9 and 10.
Data are presented as:

- mean ± SEM of 3 technical replicates in Fig. 5.
- mean ± SEM of 3 technical replicates in Fig. 6.
- mean ± SEM of 2-3 independent experiments, each performed in 3 biological replicates, in Fig. 7. The individual data points are presented in the supplement (Fig. S12). Statistical analysis was performed using the Kruskal-Wallis test, a non-parametric test for independent samples suitable for the comparison of more than two groups.
- mean ± SEM of 4 independent experiments performed in 3 biological replicates in Fig. 8a. The exact p-value (Student's t-test) is indicated in Fig. 8a.
- mean ± SD of 3 technical replicates in Fig. 8b.
- one representative experiment of two, each performed in 3 technical replicates, in Figs. 9 and 10.

Molecular Dynamics simulations: From every simulation 10.000 datapoints were sampled and an independent repeat was performed for each of the simulations. This is outlined in detail in the Materials and Methods section.

Please outline where this information can be found within the submission (e.g., sections or figure legends), or explain why this information doesn't apply to your submission:

For large datasets, or papers with a very large number of statistical tests, you may upload a single table file with tests, Ns, etc., with reference to sections in the manuscript.)

Group allocation

- Indicate how samples were allocated into experimental groups (in the case of clinical studies, please specify allocation to treatment method); if randomization was used, please also state if restricted randomization was applied

- Indicate if masking was used during group allocation, data collection and/or data analysis

Please outline where this information can be found within the submission (e.g., sections or figure legends), or explain why this information doesn't apply to your submission:

N/A; no group allocation or randomization was performed.

Additional data files ("source data")

- We encourage you to upload relevant additional data files, such as numerical data that are represented as a graph in a figure, or as a summary table
- Where provided, these should be in the most useful format, and they can be uploaded as "Source data" files linked to a main figure or table
- Include model definition files including the full list of parameters used
- Include code used for data analysis (e.g., R, MatLab)
- Avoid stating that data files are "available upon request"

Please indicate the figures or tables for which source data files have been provided:

All molecular models and simulation trajectories (Figs. 1-4) are available through the BioExcel COVID-19 Molecular Structure and Therapeutics Hub (https://covid.bioexcel.eu/simulations/).
